# A Complex Presentation of Anorexia Nervosa and Takotsubo Cardiomyopathy in a Patient of East Asian Descent

**DOI:** 10.7759/cureus.79226

**Published:** 2025-02-18

**Authors:** William M Rienas, Jingxiong Pu, Benjamin McMahon, Vivek Sarma, Rachel Silverman, Tamara Moscovich, Benedicto Borja

**Affiliations:** 1 Clinical and Translational Research, The George Washington University School of Medicine and Health Sciences, Washington, USA; 2 Psychiatry, The George Washington University School of Medicine and Health Sciences, Washington, USA

**Keywords:** anorexia nervosa, autonomy, capacity, collectivist, culture

## Abstract

Anorexia nervosa is an eating disorder characterized by severely low body weight due to psychological reasons. Its presentation and ideal management may differ depending on the cultural background of the patient. We present a 52-year-old female of East Asian descent with a unique presentation of anorexia nervosa and takotsubo cardiomyopathy. An extensive medical workup revealed no medical abnormalities, she reported negative answers to most questions posed in eating disorder screening surveys, and she wished to be discharged against medical advice despite having a BMI of 10 kg/mg^2^. Her desire to leave as well as her perception of her eating disorder were likely influenced by her collectivist culture mentality, and factoring in her cultural background was vital in managing her care. Patient autonomy vs. beneficence was considered throughout her stay, and she was determined to have the capacity to leave against medical advice two weeks after presenting. This case aims to demonstrate that providers should consider cultural components when caring for patients with eating disorders to better understand causes, treatment preferences, and guide care.

## Introduction

Anorexia nervosa (AN) is an eating disorder characterized by significantly low body weight as a result of psychological and behavioral symptoms leading to starvation. The three criteria included in the Diagnostic and Statistical Manual of Mental Disorders, Fifth Edition, Text Revision (DSM-5-TR) are: (1) restriction of energy intake relative to requirements, leading to significantly low body weight in the context of age, sex, developmental trajectory, and physical health, (2) intense fear of gaining weight or of becoming fat, or persistent behavior that interferes with weight gain, even though at a significantly low weight, and (3) disturbance in the way in which one’s body weight or shape is experienced, undue influence of body weight or shape on self-evaluation, or persistent lack of recognition of the seriousness of the current low body weight [1]. This illness manifests due to the distorted view one develops regarding their own body weight and/or shape and may result in significant impairment in one’s ability to function physically and socially [2,3]. AN typically occurs in adolescent or young adult women and is known to be one of the most challenging mental illnesses to treat leading to one the highest mortality rates among psychiatric illnesses [4]. Approximately 5% of women die within four years of diagnosis [5] The disease course is commonly chronic, causing patients to suffer for years without effective treatment [6].

The prevalence of hospitalizations due to eating disorders has increased significantly in the past decade. In the UK, annual hospital admissions in 2020 were 24,268 people, nearly an 84% increase from 2015, with a similar increase in prevalence in the US during this timeframe [7-9]. In 2018, AN’s prevalence was estimated to be around 1% of women and under 0.5% of men in high-income countries [3]. Current treatments for AN are moderately effective at best, with recovery rates under 50%. These numbers are lower for more complex presentations, highlighting the need for more robust and effective management, especially for patients with uncommon presentations [7].

In recent years, cultural considerations have provided insight into the progression and treatment of eating disorders through what may be seen as “common sense” to patients. Anthropological theories have been used in recent decades to explain AN as a consequence of one’s culture, which may contribute valuable insight into how to approach the disease depending on the patient’s background [10,11]. The three socio-cultural components that contribute the most to differences in the development and treatment of eating disorders such as AN have been identified as peers, parents, and the media [11]. The rates of eating disorders vary in eastern vs. western countries, with lower rates reported in the east [12]. This phenomenon may stem from both cultural differences and genetic tendencies. Cultural differences appear to be gaining prominence, as there are persistent cross-country variations despite genetic heterogeneity in the US. Additionally, there has been a recent increase in prevalence in Asia, coinciding with the process of westernization [12].

Cultural differences between Western and East Asian societies have been widely documented, highlighted by the holistic and collectivist nature of East Asian cultures as opposed to the more individualized approach attributed to Western cultures [13,14]. In addition, there exist notable differences in how Eastern and Western cultures perceive the consumption of food. This supports the value of understanding cultural differences in the perception and treatment of eating disorders. Current research tends to have a “Western bias,” focusing mostly on Caucasian populations, where eating disorders often emerge due to body dissatisfaction. In contrast, eating disorders in the Eastern world often manifest in relation to a fear of losing control over one’s life [15,16]. Understanding these differences as root causes of AN may aid in disease management. We present a unique case of AN in a 52-year-old female of East Asian descent, who presented with severe AN, as determined by her BMI under 10 after she collapsed due to cardiogenic shock in the setting of takotsubo cardiomyopathy, which is transient left ventricular dysfunction caused by emotional or physical stress [17].

## Case presentation

The patient was a 52-year-old female of East Asian descent with severe anorexia presenting after collapsing due to cardiogenic shock in the setting of takotsubo cardiomyopathy, diagnosed through cardiology. She was at her mother’s assisted living facility, where she also resided when she collapsed without a trigger. Takotsubo cardiomyopathy was diagnosed through her history and a transthoracic echocardiogram, which revealed systolic function severely reduced to 10%-15%, as well as akinesis of numerous regions of the anterior, septal, and apical myocardium. Her medical history included intimate partner violence, with the partner having sexually abused her and pulled out all of her teeth but is no longer in her life. She described him as a sadistic and sexual abuser who she had to run away from and has not been sexually active for 20 years. Additionally, she recovered sepsis 10 weeks prior, which was unrelated to her missing teeth. Her prior sepsis was managed at an outside hospital and was from group A strep after a dog bite, she reported that since this incident she had had a lot of anxiety and new stressors which she would not elaborate on. The patient lives with her mother in a senior living home and endorsed longstanding food insecurity for which she sometimes needs to look in dumpsters for food. In addition, she has been unemployed and cares for a disabled adult child. She reported being of a low body weight most of her life; however, her anorexia has significantly worsened, with a 20-lb weight loss since her last discharge after having sepsis 10 weeks earlier.

Upon presenting to the emergency department, the patient was hypoglycemic to 23 mg/dL, hypothermic to 33C, had a BMI of 10 kg/m^2^, and was hypotensive with a blood pressure of 68/52. Additionally, she had a heart rate of 134, oxygen saturation of 99, significant temporal wasting, and a scaphoid abdomen. She was found to have a regular heart rate and rhythm despite high pressor requirements. Upon receiving volume resuscitation, high-dose vasopressors (IV epinephrine 8 mg administered continuously at 1 microgram/min, IV norepinephrine 8 mg administered at 4 micrograms/min, and IV vasopressin 40 units administered at 0.03 units/min). The patient was hypoglycemic to 23 mg/dL and was given 50 dextrose and 20 dextrose boluses which improved sugars to over 500 prior to volume resuscitation. After receiving volume resuscitation and recovery of euglycemia/hyperglycemia with dextrose, the patient’s mental status improved.

Upon initial psychiatry consult, the patient was calm and cooperative and was aware of significant weight loss over the last several months, which was expedited after her episode of sepsis ten weeks prior. She did endorse marathon training despite low weight, however, stated her appetite dropped markedly after her sepsis ten weeks prior to a 1/10. She attributed the weight loss to her recent sepsis episode resulting in dramatically decreased appetite and loose, oily stools. She noted that she had lost 20 pounds since her sepsis, and therefore her BMI prior to infection was around 13, and still severely underweight. The patient did not endorse any symptoms of depression or PTSD, however, endorsed insomnia and she expressed a definitive desire to remain alive. She reported having grown up in Japan at a monastery, with her only support throughout her life being her mother, who was not restricting her food intake and is her surrogate medical decision-maker. The patient did not state details of her food intake prior to sepsis; however, given her low BMI prior, it must have been very low. She did not display typical eating disorder signs such as distorted body image, binging/purging, feeling fat, or guilt upon eating. The more likely etiology was originally suspected to be a metastatic biological process given a lack of complete workup at this point.

No gastrointestinal etiology was found that could be contributing to her severely low food intake. CBC, CMP, A1C, prothrombin time, and international normalized ratio were unrevealing in any non-psychiatric cause, and a physical exam of the abdomen did not reveal any guarding, masses, or hepatosplenomegaly. Immunology and serology exams did not reveal an infectious cause. Toxicology revealed no drug intoxication. She had elevated liver function tests; however, this was likely a result of her malnutrition and heart failure and not causative as they began decreasing toward discharge with improved nutrition. No pancreatic or biliary malignancy was detected on imaging. Cardiology-related tests demonstrated normal troponin 1, creatine kinase-MB, and pro-B-type natriuretic peptide, as well as the transthoracic echocardiogram findings described earlier and EKG demonstrating long QTc sinus tachycardia with premature supraventricular complexes and with frequent and consecutive premature ventricular complexes. Chest x-ray was unremarkable. Please see Tables 1, 2 for lab tests. Please note that abnormal values were determined to be a result of her malnutrition, not a cause.

**Table 1:** Lab tests used to determine the etiology of malnourishment ALT, Alanine Transaminase; AST, Aspartate Aminotransferase; BUN, Blood Urea Nitrogen; CO2, Carbon Dioxide; eGFR, Estimated Glomerular Filtration Rate; HDL, High Density Lipoprotein; LDL, Low Density Lipoprotein; RBC, Red Blood Cell; VLDL, Very Low-Density Lipoprotein

General chemistry - value (reference range)		Lipids - value (reference range)	
Glucose level	157 mg/dL (75-110)	Cholesterol	72 mg/dL (190-260)
Sodium	137 mmol/L (135-145)	HDL	68 mg/dL (36-130)
Potassium	3.5 mmol/L (3.5-5)	VLDL Chol Calc	2 mg/dL (5-40)
Chloride	102 mmol/L (95-105)	Triglycerides	10 mg/dL (10-190)
CO2	31.0 mmol/L (22.0-30.0)	LDL Direct	<30 mg/dL (0-129)
Anion gap	14 mmol/L (0-11)	General hematology - value (reference range)	
BUN	20 mg/dL (7-17)	White blood cells	10.92 x10e^3^/mcL (4.80x10e^3^-10.80x10e^3^)
BUN/creatine ratio	64 (9-20)	Hemoglobin	9.2 g/dL (12-16)
Total protein	3.5 g/dL (6-8)	Hematocrit	27.1% (37.0-47.0)
Alkaline phosphatase	258 units/L (40-125)	Mean corpuscular volume	87.7 femtoliters (80-100)
ALT	199 units/L (10-45)	Mean corpuscular hemoglobin concentration	33.9 g/dL (33.0-37.0)
AST	70 units/L (15-50)	Platelets	54 x10e^3^/mcL (130x10e^3^-400x10e^3^)
Phosphorus	3.3 mg/dL (2.5-4.5)	Immature platelet fraction	4.9% (0.9-11.0)
eGFR creatine	113 mL/min/1.73m^2^	Nucleated red blood cell #	0.00 x10e^3^/mcL
Prealbumin	3.4 mg/dL (20.0-40.0)	Lymphocytes, %	1% (21-44)
Lactic acid	4.1 mmol/L (0.5-2.2)	Eosinophils, %	0% (0-5)
Gamma-glutamyltransferase	289 units/L (10-45)	Immature granulocytes, %	0.6% (0.1-0.3)
Vitamin B12	>1,000.0 pg/mL (200-1,000)	Lymphocytes #	0.05 x10e^3^/mcL (1.00x10e^3^-4.80x10e^3^)
Cortisol	93.50 mcg/dL	Eosinophils #	0.00 x10e^3^/mcL (0-0.65x10e^3^)
Creatinine	0.5 mg/dL (0.7-1.2)	Immature granulocytes #	0.05 x10e^3^/mcL (0.01x10e^3^-0.03x10e^3^)
Calcium	7.3 mg/dL (8.5-10.5)	Bands	3% (0-10)
Albumin level	1.6 g/dL (3.5-5)	Monocytes	1% (4-9)
Total bilirubin	2.6 mg/dL (0.2-1.3)	Neutrophils	8.98 x10e^3^/mcL (1.80x10e^3^-7.00x10e^3^)
Direct bilirubin	0.3 mg/dL (0.0-0.3)	RBC morphology	Abnormal
Indirect bilirubin	1.0 mg/dL (0.0-1.1)	Anisocytosis	1+
Magnesium	1.8 mEq/L (1.5-2.0)	Polychromosia	1+
Lipase	364 units/L (25-300)	Macrocytosis	1+
Hemoglobin A1C	4.9% (0.0-6.0)	Basophilic stippling	1
Iron	47.0 mcg/dL (40-170)	Schistocytes	1+
Transferrin	<80 mg/dL (200-330)	Reticulocytes, %	5.7% (0.5-2.0)
Folate	>20.00 ng/mL (2-20)	Red blood cells	3.09 x10e^6^/mcL (4.20x10e^6^-5.40x10e^6^)
Ammonia level	29.0 mcmol/L (10-35)	Red cell distribution width	21.9% (11.5-14.5)
Haptoglobin	135 mg/dL (30-200)	Mean platelet volume	12.7 femtoliters (7.2-11.1)
Lactic dehydrogenase	468 units/L (120-246)	Neutrophils, %	97% (40-65)
Vitamin A	7.6 mcg/dL (20-60)	Basophils #	0.02 x10e^3^/mcL (0.00x10e^3^-0.20x10e^3^)
Cardiac - value (reference range)		Poikilocytosis	2+
Creatine kinase - MB	117 units/L (35-200)	Microcytosis	1+
Troponin I	<0.012 ng/mL (0.000-0.034)	Acanthocyte	2+
Pro B-type natriuretic peptide	1,230.0 pg/mL (0-300)	Echinocytes	1+
General coagulation - value (reference range)		Direct antiglobulin test	
Prothrombin time	12.1 seconds (9.4-12.5)	Direct antiglobulin test I	Negative
Partial thromboplastin time	42.4 seconds (25.1-36.5)	Protein electrophoresis/immunofixation - value (reference range)	
International normalized ratio	1.1 (0.8-1.1)	IgGe	545 mg/dL (700-1,700)
Fibrinogen	829 mg/dL (200-500)	IgM	41 mg/dL (50-300)
Thyroid - value (reference range)		IgA	109 mg/dL (70-350)
Thyroid-stimulating hormone	3.740 mc Intl units/mmL (0.4-4.7)		

**Table 2:** Further lab tests to determine the etiology of malnourishment EAEC, Enteroaggregative Escherichia coli; EIEC, Enteroinvasive Escherichia coli; EPEC, Enteropathogenic Escherichia coli; ETEC, Enterotoxigenic Escherichia coli; HIV, Human Immunodeficiency Virus; RSV, Respiratory Syncytial Virus; STEC, Shiga Toxin-Producing Escherichia coli

General immunology/serology		Toxicology	
Adenovirus	Not detected	Amphetamine	Negative
Coronavirus NL63	Not detected	Cannabinoids	Negative
Coronavirus OC43	Not detected	Methadone	Negative
Human rhinovirus/enterovirus	Not detected	Phencyclidine	Negative
Parainfluenza virus 2	Not detected	Benzodiazepine	Negative
Parainfluenza virus 4	Not detected	Cocaine	Negative
Chlamydia pneumoniae	Not detected	Opiates	Negative
Campylobacter	Not detected	Urinalysis (reference range)	
Vibrio	Not detected	Color	Yellow
EAEC	Not detected	Specific gravity	1.011 (1.003-1.035)
ETEC	Not detected	Leukocyte esterase	Trace
EIEC	Not detected	Protein	Negative
Cyclospora cayetanensis	Not detected	Ketones	Negative
Giardia lamblia	Not detected	Bilirubin	Negative
Astrovirus	Not detected	Clarity	Clear
Sapovirus	Not detected	pH	5.5 (4.5-8)
Yersinia enterocolitica	Not detected	Nitrite	Negative
HIVc	Negative	Glucose	Negative
Hepatitis A IgM	Negative	Urobilinogen	0.2 Ehrl units/dL (0.2-1)
Hepatitis B surface antigen	Negative	Blood	Small mg/L
Flu A PCR	Negative	White blood cells	4/HPF (0-4)
Influenza A	Not detected	Bacteria	None seen
RSV	Not detected	Mucous	Present
Coronavirus HKU1	Not detected	Hyphae yeast	Moderate
Coronavirus 229E	Not detected	Calcium oxalate crystal	Present
Human metapneumovirus	Not detected	Red blood cells	877/HPF (0-2)
Parainfluenza virus 1	Not detected	Squamous epithelial cells	1/HPF (0-4)
Parainfluenza virus 3	Not detected	Budding yeast	Present
Bordetella pertussis	Not detected	Hyaline casts	>20/LPF (0-2)
Mycoplasma pneumoniae	Not detected	Chloride	130 mmol/L
Plesiomonas shigelloides	Not detected	Sodium	87.0 mmol/L
Vibrio cholerae	Not detected	Osmolality	399 mOsmol/kg (50-1,200)
EPEC	Not detected	Potassium	39.9 mmol/L
STEC	Not detected	Urea	252 mg/dL (90-230)
Cryptosporidium	Not detected	Culture stool	
Entamoeba histolytica	Not detected	Salmonella	Negative
Adenovirus F 40/41	Not detected	Campylobacter	Negative
Norovirus GI/GII	Not detected	Yersinia	Negative
Rotavirus A	Not detected	Serology continued	
Hepatitis B Core IgM	Negative	Antinuclear antibodies	Negative
Hepatitis C antibody	Negative	Selenium	51 mcg/L (110-165)
Flu B PCR	Negative	Zinc	42 mcg/dL (80-120)
Influenza B	Not detected	Vitamin B1	242.4 nmol/L (74-222)
Clostridioides difficile Ag	Positive	Tumor markers	
Clostridioides difficile toxin	Negative	CA 19-9	33.1 U/mL (0-37)
Bordetella parapertussis	Not detected		
Salmonella	Not detected		

After a nasogastric feeding tube was placed, she was noted to have one to two bowel movements per day, no steatorrhea, visual soft/brown stools, and some watery stools mixed with urine. The patient and her mother self-removed the nasogastric tube 10 days after placement due to discomfort, and anorexia was proposed as the etiology of her weight loss. AN was proposed given her restriction of energy intake relative to requirements leading to a significantly low body weight, and her denial of the seriousness of the current low weight. The patient expressed motivation to eat and had significant improvements in electrolytes and glucose levels upon feeding. She was completing 30%-50% of her meals and supplemented her diet with protein shakes. Her blood pressure stabilized with systolic pressure in the 120s off of vasopressors, however, her cardiomyopathy remained a significant issue with an ejection fraction of 10%-15%.

Approximately one week after arrival, the patient remained in the intensive care unit and had a desire to leave against medical advice. Psychiatry was consulted to assess for capacity to make medical decisions. As detailed in Appelbaum’s criteria, this involves an interview with patients to assess their communication of a medical choice, an appreciation of the relevant facts contextual to that choice, reasoning for making said choice, and an appreciation of the consequences [18]. The consult psychiatry team communicated with the patient in English and her mother in Mandarin via a phone interpreter. The patient had a consistent desire to leave with an understanding that her illness was likely fatal if left untreated, but a steadfast belief that her parent’s love would prevent death from occurring. She described a desire to die at home, explained that care by her mother at home is best, and expressed that the medical team could not adequately treat her. Additionally, she was concerned that she needed to care for her mother and daughter. The patient’s mother explained that both of them would die soon regardless, and therefore, the patient should be discharged. The mother explained that the patient’s condition has been occurring for years and expressed a strong desire for them to die together in peace. A lack of capacity was determined due to a lack of insight by the patient into the risks of leaving the hospital and not providing a rationale that justified the risk of imminent death. Additionally, if the patient and her mother believe that she will inevitably die soon, this understanding was incorrect as there was still an ongoing workup for the cause of malnutrition, and she does not have a diagnosed terminal illness. Upon the determination that the patient could not leave, the patient stated she would kill herself that night in the hospital. At that time, the patient was started on mirtazapine 7.5mg orally once daily due to a potential eating disorder and insomnia.

Two days later, psychiatry conducted another capacity consultation. The patient and her mother retained their desire and reasons for leaving against medical advice. Additionally, the patient’s suicidal thoughts were no longer present as she claimed to be religious and needed to care for her mother and daughter. Psychiatry was able to speak with both the patient and her mother directly due to bilingual staff. The patient’s mother repeated her reasons for the patient to leave and added that they would prefer to die at home due to their cultural background. Notably, the patient and her mother did not feel that death would be imminent due to medical causes, rather they felt it could happen at any time because of religious reasons. Additionally, the mother explained their family dynamic, stating that the patient takes care of both her, due to language barriers, and her disabled daughter. Therefore, she would not commit suicide nor stop caring for them due to cultural reasons; however, if the patient remained in the hospital, it would “kill all three” of them.

The patient endorsed a feeling of being covered in her feces and urine due to impaired intestinal absorption while in her bed in the hospital. However, upon checking, there was no feces or urine present. The patient was calm and cooperative with good eye contact. However, she was again determined to not have the capacity to leave against medical advice due to her lack of insight into the risk of imminent death and not providing a rationale that justifies the risks. The patient and her mother had some understanding that the patient would not live long and would rather die at home, and this had a strong religious and cultural component; however, the patient did not have a terminal illness.

Screening questions for an eating disorder, such as feeling fat, guilt upon eating, binging/purging, and preoccupations with body image, were negative. However, the patient was a previous marathon runner and was continuing to train four to five times a week but stopped after her sepsis episode 10 weeks prior due to fragility and stated that eating is a “pleasure.” The patient felt that the way for her to improve was to move around, not necessarily to eat. This led us to suspect an abnormal presentation of an eating disorder with a strong religious and cultural component.

The next day, approximately two weeks after arrival, psychiatry was consulted to determine the capacity to leave against medical advice. The patient and her mother were both present and consistent in their desire to leave. The patient and her mother both understood the risks of going home and that it may result in imminent death, and the mother insisted that if the patient does not go home her entire family will die. The patient expressed no desire to get better and did not want more active treatment but was open to palliative care. The patient again complained of being covered in feces, which was not present, and a delusion and was fixated on being cleaned. The patient appeared to not have insight about the possibility of having an eating disorder; however, she could communicate her choice, knew her current medical problem/treatment plan, and acknowledged treatment benefits and the risks of going home. The patient and her mother were aware that if the patient’s malnutrition persists, she will medically decompensate and die soon. Additionally, the patient stated that she lives minutes away and can return if necessary. Psychiatry determined that the patient could leave against medical advice. Extensive workup for nonpsychiatric causes of malnutrition was completed and no medical causes were identified. A joint meeting was held between psychiatry, palliative care, the patient and her mother, and primary care to align her care with her goals later that day and the patient was discharged. During this joint meeting, the patient acknowledged her malnourishment and heart condition, but did not know what it meant that her heart condition could not get better given her nutritional status. She felt her care is better at home where she is close by and can return for appointments. She again expressed understanding of the risks of going home, stating that “if death is my fate, I accept it,” denied suicidal ideation, and denied any hallucinations. She was determined to have capacity to leave given her understanding, ability to discuss her medical condition, and understanding of options and alternative treatments.

The patient had a BMI of 10 kg/mg^2^ upon discharge and was instructed to attend a follow-up appointment within three to five days to ensure she was receiving appropriate palliative or hospice care. Additionally, the patient was prescribed mirtazapine and given educational materials regarding her conditions and diet recommendations. It is unclear the level of care she can receive while living in her mother’s assisted living facility; however, she was receptive to additional support in an outpatient setting such as palliative care and hospice and provided information on how to access this. The patient was lost to follow-up.

## Discussion

We present a case of AN with an abnormal presentation and strong cultural component. The 52-year-old female patient had no history of eating disorders and did not display typical signs such as distorted body image, binging/purging, feeling fat, or guilt upon eating. She met the DSM-5-TR criteria for AN given the wide range of possible presentations [1,19]. Specifically, she had a restriction of energy intake relative to requirements, leading to a significantly low body weight in the context of her age, sex, and physical health, she had persistent behavior that interferes with weight gain, even though at a significantly low weight, and she had a lack of recognition of the seriousness of the current low body weight. Ultimately, the patient was discharged against medical advice given her capacity.

Understanding how different cultures contribute to the perception of multiple health issues, including eating disorders, may help in treatment management. This patient came from a family highly dependent on each member. It is widely believed that East Asian cultures are more collectivistic, and Western cultures are more focused on individualism [20,21]. In collectivist cultures, the self is viewed as an interdependent part of the group, and members place group concerns above personal matters. This contrasts with individualistic cultures, where members more often place individual concerns over that of the group [22]. IWhen this patient insisted on leaving, we saw her prioritize the interests of her group, which included her mother and daughter, who were heavily reliant on her, over her own. Her mother needed the daughter to translate for her as she was not a native English speaker, and the patient’s daughter had an intellectual disability and relied on the family to support her. The extreme dependence did not run in a single direction; this patient relied heavily on her family to keep her alive given her current deteriorating health status, and they all lived together in the grandmother’s assisted living facility. The patient’s mother directly stated on multiple occasions that all three would die if the patient remained in the hospital. Additionally, this patient and her mother were both content with dying at peace around the same time, further demonstrating a collectivist mindset where death together is acceptable over focusing on individual needs in the hospital. An understanding of what this patient values was necessary in providing medical treatment and played a role in determining her capacity.

The patient’s prior trauma likely contributed to the development of AN, as she was heavily abused by her husband in the recent past. She has been under extreme emotional or physical stress, widely known to contribute to the development of takotsubo cardiomyopathy, which carries the colloquial name “broken heart syndrome” [17,23]. It is unclear how this contributed to her development of anorexia, however, the patient presented with no teeth, and there may be some connection to eating given this. Regardless, the patient will need her cardiomyopathy to resolve, which will only occur with adequate nutrition and resolution of her acute stressors. She was informed of this necessity and provided resources such as how to access further medical care and therapy options. Her delusions regarding sitting in diarrhea and urine given their absence in the hospital may be a form of rationalization for not eating. It is possible that her previous episodes of sepsis contributed to her development of anorexia, as there is some older evidence of a similar effect in which streptococcus infection, particularly group A strep, triggers obsessive-compulsive disorder [24-26], and her prior sepsis was from group A strep. However, an extensive workup revealed no non-psychiatric etiology of anorexia.

This patient did not want care in the hospital and wanted to leave. Her surrogate medical decision-maker agreed with this and wanted the patient to leave against medical advice. There was an ongoing ethical question of beneficence vs. patient autonomy throughout the patient’s stay, which was posed to multiple treatment teams. Capacity can wax and wane and given her demonstration of capacity at one point in her stay, the patient was allowed to be discharged, ultimately favoring patient autonomy over beneficence despite her consistent BMI of 10 kg/mg^2^. Even with the best current treatment guidelines, eating disorder recovery rates fall below 50%, and keeping this patient in the hospital against her and her family’s will would likely lead to less willingness to seek further medical help in the future [7,27]. At the time of discharge, the patient was very willing to return for medical appointments if needed, open to palliative care, provided appropriate medications for insomnia and anorexia (mirtazapine 7.5mg oral tablet once daily), and provided information on how to seek further medical care with a recommendation for follow-up in three to five days.

## Conclusions

In our case report, we present a 52-year-old female of East Asian descent with AN and takotsubo cardiomyopathy presenting after collapsing in the setting of cardiogenic shock. The patient had a BMI of 10 kg/mg^2^ and wished to be discharged against medical advice. She was determined to have capacity two weeks after presenting and allowed to leave. Her presentation was unique giving negative answers to most questions posed in eating disorder screening surveys. However, extensive medical workup revealed no medical abnormalities. The main finding in this case report is that determining the capacity to make medical decisions, such as leaving against medical advice, is largely impacted by one’s cultural background, and to optimize care physicians need to account for how various cultures weigh different priorities in making medical decisions. This patient's strong collectivist and spiritual background provided the context to determine she could leave against medical advice and guide her care even given her severely low BMI and cardiomyopathy. Incorporating cultural perspectives into the management of AN is necessary for improving treatment outcomes. More broadly, understanding and addressing cultural factors can enhance diagnostic accuracy, patient engagement, and treatment effectiveness. To optimally impact patients, clinicians should adopt culturally sensitive approaches to better align treatment with patients’ values and beliefs, ultimately fostering positive mental and physical health and emphasizing the importance of lifestyle and cultural considerations in psychiatric practice. This work adds to a growing body of evidence showing different manifestations of eating disorders across cultures and how to optimally treat them.

## References

[1] (2025). Diagnostic and Statistical Manual of Mental Disorders, Fifth Edition, Text Revision. https://www.mredscircleoftrust.com/storage/app/media/DSM%205%20TR.pdf.

[2] Solmi M, Wade TD, Byrne S (2021). Comparative efficacy and acceptability of psychological interventions for the treatment of adult outpatients with anorexia nervosa: a systematic review and network meta-analysis. Lancet Psychiatry.

[3] Zipfel S, Giel KE, Bulik CM (2015). Anorexia nervosa: aetiology, assessment, and treatment. Lancet Psychiatry.

[4] Smink FR, van Hoeken D, Hoek HW (2012). Epidemiology of eating disorders: incidence, prevalence and mortality rates. Curr Psychiatry Rep.

[5] Auger N, Potter BJ, Ukah UV (2021). Anorexia nervosa and the long-term risk of mortality in women. World Psychiatry.

[6] Crow SJ (2023). Terminal anorexia nervosa cannot currently be identified. Int J Eat Disord.

[7] Downs J, Ayton A, Collins L (2023). Untreatable or unable to treat? Creating more effective and accessible treatment for long-standing and severe eating disorders. Lancet Psychiatry.

[8] Thompson KA, Bauman V, Sunderland KW (2023). Incidence and prevalence of eating disorders among active duty US military-dependent youth from 2016 to 2021. Int J Eat Disord.

[9] Daly M, Costigan E (2022). Trends in eating disorder risk among U.S. college students, 2013-2021. Psychiatry Res.

[10] Eli K, Warin M (2018). Anthropological perspectives on eating disorders: deciphering cultural logics. Transcult Psychiatry.

[11] Munro C, Randell L, Lawrie SM (2017). An integrative bio-psycho-social theory of anorexia nervosa. Clin Psychol Psychother.

[12] Pike KM, Dunne PE (2015). The rise of eating disorders in Asia: a review. J Eat Disord.

[13] Liu C, Li J, Chen C, Wu H, Yuan L, Yu G (2021). Individual pride and collective pride: differences between Chinese and American corpora. Front Psychol.

[14] Fan G, Carlson KD, Thomas RD (2021). Individual differences in cognitive constructs: a comparison between American and Chinese culture groups. Front Psychol.

[15] Monocello LT, Dressler WW (2022). Cultural consonance, body image, and disordered eating among young South Korean men. Soc Sci Med.

[16] Pearcey SM, Zhan GQ (2018). A comparative study of American and Chinese college students' motives for food choice. Appetite.

[17] Ghadri JR, Cammann VL, Napp LC (2016). Differences in the clinical profile and outcomes of typical and atypical Takotsubo syndrome: data from the International Takotsubo Registry. JAMA Cardiol.

[18] Bari BA, Beach SR (2024). Evaluating capacity: Appelbaum’s framework interpreted diagrammatically. J Acad Consult Liaison Psychiatry.

[19] Feltner C., Peat C., Reddy S. (2024). Appendix A Table 1, Summary of DSM-5 Diagnostic Criteria for Eating Disorders. Screening for Eating Disorders in Adolescents and Adults: An Evidence Review for the U.S. Preventive Services Task Force.

[20] Hornsey MJ, Greenaway KH, Harris EA, Bain PG (2019). Exploring cultural differences in the extent to which people perceive and desire control. Pers Soc Psychol Bull.

[21] Abramov DM, Peixoto PC (2022). Does contemporary Western culture play a role in mental disorders?. Front Psychiatry.

[22] Jiao J, Zhao J (2023). Individualism, collectivism, and allocation behavior: evidence from the ultimatum game and dictator game. Behav Sci (Basel).

[23] Assad J, Femia G, Pender P, Badie T, Rajaratnam R (2022). Takotsubo syndrome: a review of presentation, diagnosis and management. Clin Med Insights Cardiol.

[24] Sokol MS, Gray NS (1997). Case study: an infection-triggered, autoimmune subtype of anorexia nervosa. J Am Acad Child Adolesc Psychiatry.

[25] Sokol MS (2000). Infection-triggered anorexia nervosa in children: clinical description of four cases. J Child Adolesc Psychopharmacol.

[26] Descalço N, Gomes RD, Maia A, Barahona-Corrêa B, Oliveira-Maia A, Oliveira J (2023). Infections and obsessive-compulsive disorder - results from a systematic review and meta-analysis. Eur Psychiatry.

[27] Frostad S, Bentz M (2022). Anorexia nervosa: outpatient treatment and medical management. World J Psychiatry.

